# Digital Disparities Among Older Adults With and Without Disabilities in the U.S.: An Examination of the Census Data

**DOI:** 10.1093/geroni/igaf122.2585

**Published:** 2025-12-31

**Authors:** Xiayu Chen, Kang Sun

**Affiliations:** University of Central Florida, Urbana, Illinois, United States; Southern Illinois University, Carbondale, Illinois, United States

## Abstract

The rapid expansion of digital technologies has significantly reshaped the ways in which older adults communicate, access resources, and maintain independence. Despite federal efforts such as the Digital Equity Act and the Affordable Connectivity Program, substantial disparities persist, especially among older adults with disabilities. Using nationally representative data from the 2023 Current Population Survey (N = 21,152), this study investigates digital access, usage patterns, and barriers. Weighted descriptive and logistic regression analyses identified significant disparities. Among older adults, 28.77% have disabilities, and only 73.5% reported home-based digital technology use compared to those without disabilities. Smartphones are the primary device, primarily used for communication, social networking, e-commerce, financial management, and health applications. However, digital exclusion remains a pressing issue, with 26.5% of older adults with disabilities lacking home technology access versus 16.71% of those without disabilities. The most common barriers include lack of interest, financial constraints, and accessibility issues. Logistic regression demonstrated significantly lower likelihood of home digital access for older adults with disabilities (OR = 0.55, p < .001), particularly among female, economically and educationally disadvantaged individuals, and rural residents. These findings highlight the urgent need for targeted policy and community interventions, emphasizing expanded affordability programs, improved technological accessibility, and tailored digital literacy initiatives. Addressing these barriers at multiple levels will be essential for fostering digital equity and inclusion among older adult populations in the U.S.

